# Vitamin A, C and/or E Intake During Pregnancy and Offspring Respiratory Health: A Systematic Review and Meta‐Analysis

**DOI:** 10.1111/jhn.70086

**Published:** 2025-07-02

**Authors:** Vanessa E. Murphy, Megan E. Jensen, Soriah Harvey, Tesfalidet Beyene, Jake Gregson, Farihah Islam, William Huang, Katie Aistrope, Adam Collison

**Affiliations:** ^1^ School of Medicine and Public Health University of Newcastle Callaghan New South Wales Australia; ^2^ Asthma and Breathing Research Program Hunter Medical Research Institute Newcastle New South Wales Australia

**Keywords:** antioxidants, childhood, maternal, pregnancy, respiratory, vitamins

## Abstract

**Introduction:**

Poor respiratory health in childhood is common, and asthma is the most common chronic disease among children, for which there is no known cure. Maternal intake of vitamins (A, C, E) may be a modifiable nutritional exposure to reduce adverse respiratory health in offspring.

**Objective:**

We aimed to systematically review the evidence for the association between maternal vitamin (A, C, E) intake during pregnancy (via questionnaire or blood assay) and respiratory outcomes in the offspring.

**Methods:**

Studies identified through electronic databases were eligible if they assessed maternal levels and/or intake of vitamins A, C and/or E via dietary intake or supplements in pregnancy and respiratory outcomes in the first 5 years of life. Meta‐analyses were conducted where possible. Outcomes included wheeze, cough, asthma, infant respiratory distress syndrome (RDS), respiratory tract infection (RTI) and lung function measurement.

**Results:**

Of 1170 articles screened, 12 observational studies and six RCTs met the inclusion criteria (total sample size *n* = 58,769). Meta‐analysis could not be performed for vitamin A; however, there was no evidence to suggest that maternal vitamin A intake improves early life respiratory outcomes in offspring. Two RCTs found that vitamin C supplementation (500 mg/day vs. placebo) reduced the incidence of wheeze at 12 months (*n* = 206 children) and 5 years (*n* = 213 children) in pregnancies exposed to smoking. In meta‐analyses, maternal intake in the highest vitamin E quartile versus lowest reduced the odds of wheeze at 2 years by 36% (aOR: 0.64, 95% CI: 0.47–0.87, *n* = 2 observational studies, very low certainty); this was not true for vitamin C intake (aOR: 0.85, 95% CI: 0.63–1.16, *n* = 2 observational studies, very low certainty). Vitamin supplementation (C + E) was not associated with infant RDS (OR: 1.15, 95% CI: 0.80–1.64, *n* = 2 studies, moderate certainty) relative to placebo.

**Conclusion:**

There may be some benefit to vitamin C supplementation during pregnancy in the context of maternal smoking or higher maternal vitamin E intake during pregnancy, for reducing the risk of childhood wheeze in early life. This emerging evidence warrants further studies to enable translation into dietary guidelines.

## Summary

- There was no evidence to suggest that maternal vitamin A intake improves early life respiratory outcomes in offspring; however, meta‐analyses were not possible.
- Two RCTs found that vitamin C supplementation (500 mg/day vs. placebo) reduced the incidence of wheeze in infants and children in pregnancies exposed to smoking.
- Vitamin C + E was not associated with improved respiratory health; however, the highest vitamin E intake (vs. the lowest quartile) was associated with reduced wheeze at 2 years.

## 1 Introduction

Respiratory illness in early childhood causes a significant burden to Australia's healthcare system, with one in three children affected by wheeze in the first 3 years of life [1]. While there is considerable phenotypic heterogeneity [1], wheeze in early life, especially with viral infection, as each is independently associated, is a strong predictor for subsequent childhood asthma [2]. Asthma is the most common chronic disease among children, for which there is currently no known cure [3]. While the underlying causes of asthma are not yet fully understood, it is likely that the risk can be modified while the lungs are developing, by environmental exposures in pregnancy and early life [3]. These exposures include nutritional exposures, which are modifiable and potential targets for future clinical trials.

The antioxidant theory hypothesises that the increased prevalence of asthma is a combination of both increased air pollution and a decrease in antioxidant intake in modern western diets [4, 5]. This is believed to result in increased susceptibility to oxidant damage and inflammation of the developing lungs, which is known to be a factor in asthma development [6, 7].

Vitamin A is a fat‐soluble vitamin known to play a protective role in the cells of the respiratory system, particularly against oxidation [8]. However, vitamin A deficiency is a public health problem affecting 19 million pregnant women worldwide [9]. Research has found that, in patients with chronic obstructive pulmonary disease (COPD), those with a higher concentration of serum retinol, a form of vitamin A, maintained improved respiratory function [10]. Furthermore, maternal vitamin A status may be an important determinant of embryonic alveolar formation [11], and vitamin A deficiency in a mother during pregnancy could have lasting adverse effects on the lung health of her offspring, such as decreased lung function in childhood (9–13 years) [12]. However, a 2023 systematic review found that vitamin A intake in pregnancy was positively associated with current risk of asthma at age 7, making the protective relationship between maternal vitamin A and childhood respiratory health questionable; however, only two studies were included in the meta‐analysis, and no other ages or outcomes were examined. This highlights that few human studies have actually examined the risk of childhood respiratory outcomes in relation to maternal vitamin A, indicating a meta‐analysis would be beneficial to pool all current data in young children (0–5 years of age) to clearly answer research questions of interest [13].

Vitamin C is a water‐soluble vitamin that plays an important role in maintaining the antioxidant capacity of the cell, particularly within the cytoplasm [14]. The role of vitamin C in lung health has been the subject of many studies over the last two decades; however, the results are mixed as to whether or not vitamin C intake positively affects respiratory health. Some studies have shown that there is a positive relationship between vitamin C intake during pregnancy or in a general population and lung function [14, 15], while others have shown an inverse relationship [16]. Recently, a large Cochrane review on the use of vitamin C for asthma, including studies in adults and children ( > 5 years), concluded that there was insufficient evidence to support its use and further, more robust studies were required [17]. Children under 5 years were not examined in this review, and the use of vitamin C in pregnancy as a protective factor for the lung health of the fetus was also not examined.

Vitamin E, as well as being a potent antioxidant, plays a role in the body's cell‐mediated immune responses, particularly with immunoglobulin E (IgE) levels [17, 18]. Vitamin E is a fat‐soluble vitamin that humans require regular dietary intake to maintain adequate stores [18]. Studies have demonstrated that an increase in vitamin E intake, and/or its various precursors, improves lung function across the lifespan, while an insufficiency of vitamin E can lead to increased airway inflammation [5, 17]. Some studies have identified low vitamin E blood levels during pregnancy to be associated with increased respiratory issues such as wheeze and recurrent wheeze in children [17, 18]. A systematic review was conducted on vitamin E (serum level or supplementation during pregnancy or childhood) and found that maternal vitamin E intake was negatively associated with asthmatic diseases in offspring, regardless of sample size and follow‐up length (*n* = 10 studies, odds ratio [OR]: 0.74, 95% CI: 0.61–0.89) [5], however, specific levels or cut offs of vitamin E were not examined due to heterogeneity in the studies, and populations included children up to 18 years old, making the effect in young children (0–5 years) impossible to know. Several studies have also been published since this review, examining maternal vitamin E and offspring respiratory outcomes [19, 20, 21].

Vitamin supplementation for pregnant women to improve the respiratory outcomes of their infants is a growing area of interest and there is an abundance of literature related to vitamin D. However, less is known about the potential of antioxidant vitamins A, C and E in promoting respiratory health in early life (0–5 years). The aim of this systematic review is to synthesise the literature on the association between maternal intake and supplementation of vitamins A, C and/or E during pregnancy and offspring respiratory outcomes in the first 5 years of life.

## 2 Materials and Methods

### 2.1 Protocol and Registration

The protocol was registered with the PROSPERO International Prospective Register of Systematic Reviews on 23 June 2021 (CRD420212544327). The protocol was developed in line with the Preferred Reporting Items for Systematic Reviews and Meta‐Analyses (PRISMA) and Meta‐analysis of Observational Studies in Epidemiology (MOOSE) guidelines.

### 2.2 Search Strategy

A literature search was conducted using the electronic databases MEDLINE, Cochrane, EMBASE and CINAHL. Search terms included those related to pregnancy (pregnancy, gestation and obstetric), vitamin A (vitamin A, retinol), vitamin C (vitamin C, ascorbic acid), vitamin E (vitamin e, tocopherol) and respiratory (asthma, wheeze, newborn respiratory distress syndrome [RDS], respiratory function tests, respiratory hypersensitivity, respiratory sounds, respiratory tract diseases, pneumonia, bronchiolitis, tidal breathing, multiple breath washout, forced oscillation technique, croup and transient tachypnoea of the newborn).

The search was limited to human studies published from January 2001 to November 2024, available in the English language. This date was chosen to include contemporary literature.

### 2.3 Inclusion and Exclusion Criteria

Studies were included if they were randomised controlled trials (RCTs), cross‐sectional, case–control and cohort studies of pregnant women where maternal vitamin A, C and/or E intake or levels were measured or supplements were given during pregnancy, and data were available on at least one respiratory outcome for their offspring up to 5 years of age. Outcomes included wheeze, cough, asthma, infant RDS, respiratory tract infection (RTI) and lung function measurement.

Studies were excluded if they did not contain primary data or if maternal vitamin levels or intake were only measured pre‐conception or postpartum. Conference papers and review articles were excluded.

Titles and abstracts were screened by two independent reviewers using Covidence, followed by full‐text screening. Any conflicts were discussed with a third reviewer until consensus was reached.

### 2.4 Data Extraction

Data were extracted into an electronic database by one reviewer and confirmed by a second reviewer. Extracted data included: author and country, study design, population, age at follow‐up, outcome/s, maternal vitamin intake/intervention, assessment of maternal vitamin intake and/or level, main result/s and quality score or risk of bias assessment.

### 2.5 Quality Assessment

Quality assessment of included studies, including publication bias, was conducted using the Newcastle Ottawa Scale (NOS) for cohort studies and the Cochrane Risk of Bias (RoB) tool for RCTs [22, 23]. The NOS assesses quality based on three domains: selection, comparability and exposure, and has a maximum score of 9, with a score of 8–9 meaning 'very good' quality, 6–7 meaning 'good', 4–5 meaning 'satisfactory' and 0–3 meaning 'unsatisfactory.' The Cochrane RoB assesses risk of bias across five domains (selection, performance, attrition, reporting and other) and provides a categorical judgement (high, low or unclear) based on the outcome scores. Two reviewers assessed quality and any disagreements were discussed with a third reviewer.

## 3 Analysis

Narrative synthesis described the findings according to maternal vitamin intake or supplementation. Where data from two or more studies could be combined (due to examining the same age or age range, and the same outcome measure), we conducted meta‐analysis following standard methodological guidelines using Review Manager 5.4 [24]. The odds ratio of the offspring respiratory outcomes according to maternal vitamin intake or supplementation was calculated using the inverse variance method. Adjusted and unadjusted models were used where appropriate. Heterogeneity between studies was assessed using the *χ*² test (with *p* < 0.1 indicating significant heterogeneity) and *I*² parameter (where *I*² > 60% indicates moderate heterogeneity) [25]. We used Grading of Recommendations, Assessment, Development and Evaluations (GRADE) [26] to determine the certainty of the meta‐analysis results.

## 4 Results

### 4.1 Search Results

**Figure 1:**
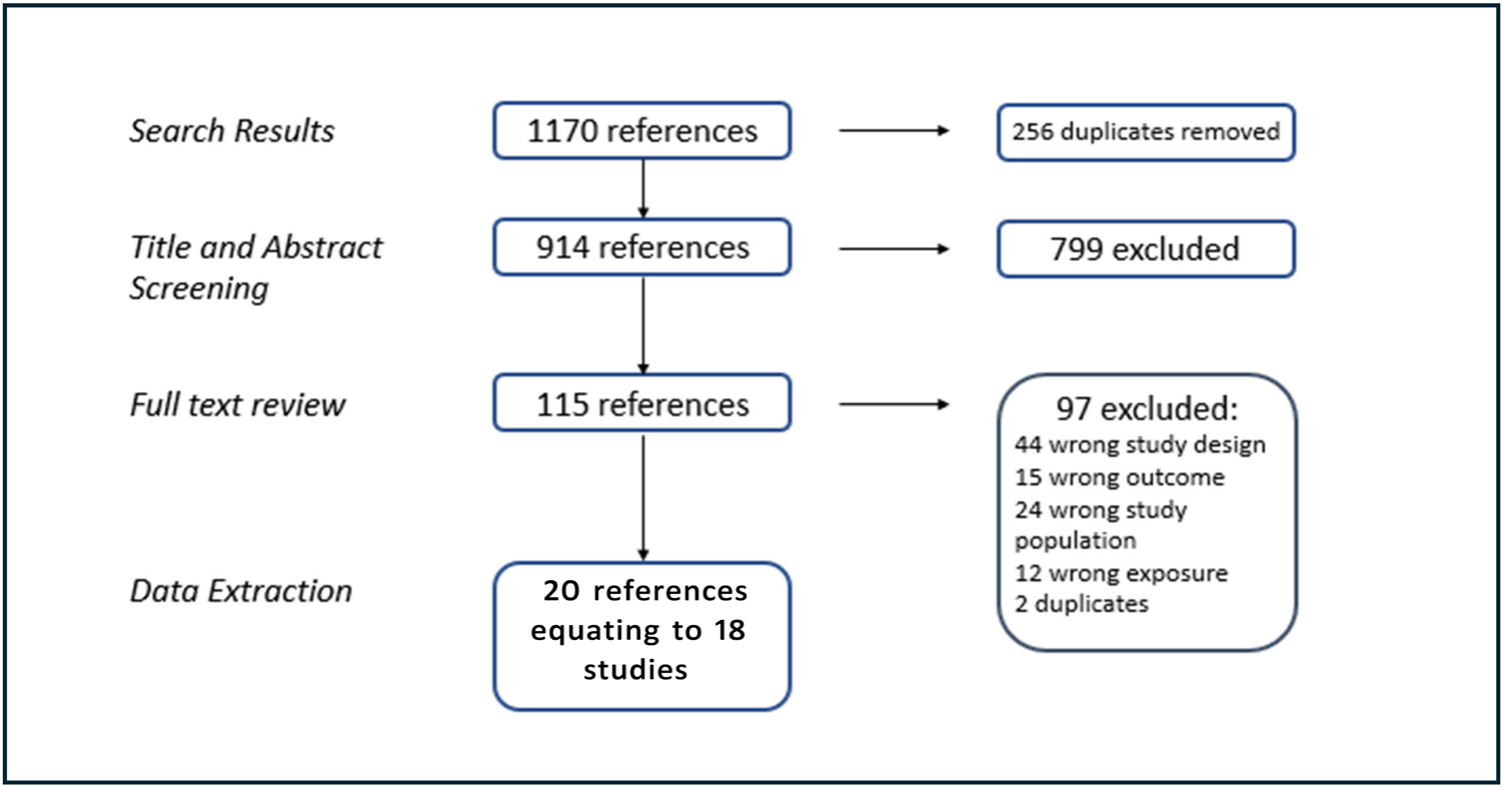
PRISMA flow diagram of included studies.

The search yielded 1267 articles, reducing to 914 after removal of duplicates. Full‐text screening was conducted on 147 articles. Twenty articles, equating to 18 studies, were included in the final review (Figure 1 and Table 1).

**Table 1:** Characteristics of included studies.

Author & country	Study design	Population	Age at follow‐up	Outcome/s	Assessment of outcome/s	Maternal vitamin intake/intervention	Assessment of maternal vitamin intake and/or level	Main result/s	Quality score or risk of bias assessment
Borna et al. [27] Iran	RCT	Pregnant women with PPROM at 26–34 weeks gestation *N* = 60	Birth	Respiratory distress syndrome (RDS)	Medical records	Intervention group: Vitamin C (500 mg) + Vitamin E (400IU/daily) Control group: placebo	Not measured	RDS Treatment versus control group 15/30 versus 15/30 *p*‐value: 0.27	Low RoB
Fawzi et al. [28] Tanzania	RCT	788 mother–infant pairs	2 years	Cough with rapid respiratory rate	Medical records	Intervention group: Vitamin A (and multivitamin including vitamins C and E) throughout pregnancy from recruitment (12–27 weeks) 4 regimes: 5000IUA+30 mg Betacarotene Multivitamin no Vitamin A Multivitamin + Vitamin A (as above) Placebo	Not measured	Cough with a rapid respiratory rate (relative risk, 95% CI) Vitamin A recipients: 0.69 (0.49, 0.96) Multivitamin only recipients: 1.08 (0.75, 1.55)	Low RoB
Greenough et al. [29] UK	RCT	752 mother–child dyads	2 years	Wheeze & asthma	Questionnaire	Intervention group: Vitamin C (1000 mg) + Vitamin E (400 mg/daily) Control group: placebo	Not measured	Asthma in first 12 months (odds ratio, 99% CI) Asthma:0.94 (0.42, 2.11) *p*‐value: 0.855 Wheeze in first 12 months (odds ratio, 99% CI) Wheeze ever: 0.78 (0.50, 1.21) Wheeze > once/week: 0.81 (0.32, 2.06) Wheeze in second 12 months Wheeze ever: 0.97 (0.62, 1.52) Wheeze > once/week: 0.83 (0.26, 2.59)	Low RoB
Hauth et al. [30] USA	RCT	698 mother–infant pairs	Birth	RDS	Medical record	Intervention group: Vitamin C (1000 mg) + Vitamin E (400 mg) Control group: placebo	Not measured	RDS, *n* (%) Intervention: 56 (18.6%) Placebo: 49 (17.3%) (*p* = 0.67)	Low RoB
aMcEvoy et al. [15] USA	RCT	206 pregnant women who smoke	3 and 12 months	wheeze	Questionnaire	Intervention group: Vitamin C (500 mg/day) Control group: placebo	Fasting plasma ascorbic acid (Vitamin C) levels (28–30 weeks gestation)	Incidence of wheeze at 12 months (*n*, %) Intervention: 51 (43.2%) Placebo: 63 (53.9%)	Low RoB
McEvoy et al. [31] USA	RCT	251 pregnant women who smoke	Newborn (within 72 h) and 12 months	wheeze	Questionnaire	Intervention group: Vitamin C (500 mg/day) Control group: placebo	Fasting plasma and urine ascorbic acid (Vitamin C) levels (28–30 weeks gestation)	Incidence of wheeze at 12 months (*n*, %) Intervention: 15 (21%) Placebo: 31 (40%) adjusted relative risk 0.56 (95% CI 0.33, 0.95)	Low RoB
^a^McEvoy et al. [32] USA	RCT	251 pregnant smokers and 213 children	5 years	Current wheeze	Questionnaire	Intervention group: Vitamin C (500 mg/day) Control group: placebo	Plasma ascorbic acid level	Current wheeze (adjusted OR, 95% CI) 0.41 (0.23, 0.74)	Low RoB
^a^Martindale et al. [33] Scotland	Cohort	1300 mother–infant pairs	13–24 months	Ever wheeze	Questionnaire	Vitamin A precursor (beta carotene) Vitamin C Vitamin E	FFQ (34 weeks gestation)	Results not reported for Vitamin A—Study reports all levels nonsignificant association for wheeze. Ever wheeze Vitamin C (adjusted OR, 95% CI) Quintile (Q)1: 1.00 Q2: 1.79 (1.00, 3.19) Q3: 2.50 (1.45, 4.39) Q4: 1.52 (0.83, 2.77) Q5: 2.25 (1.26, 4.02) *p*‐trend= 0.034 Vitamin E (adjusted OR, 95% CI) Q1: 1.00 Q2: 0.73 (0.44, 1.21) Q3: 0.70 (0.42, 1.44) Q4: 0.71 (0.43, 1.18) Q5: 0.79 (0.47, 1.31) *p*‐trend= 0.363	NOS 6
Maslova et al. [34] Denmark	Cohort	44,594 mother–infant pairs	18 months	Wheeze	Interview	Vitamins A, E and K	FFQ (25 weeks gestation)	Any wheeze (aRR, 95% CI) Vitamin A (from supplements, not diet) quintiles 1:Ref 2:1.04 (0.96–1.12) 3:1.02 (0.94–1.11) 4:0.98 (08–1.07) 5:1.02 (0.93–1.11) Vitamin E (from diet) quintiles 1:Ref 2:0.98 (0.90–1.07) 3:0.97 (0.89–1.06) 4:1.01 (0.91–1.11) 5:1.01 (0.91–1.12)	NOS 6
Miyake et al. [4] Japan	Cohort	1002 pregnant women 763 mother–infant pairs	16–24 months	Wheeze	Questionnaire	Vitamin A (alpha and beta carotene) Vitamin C Vitamin E	FFQ (assessing diet 1 month before recruitment)	Wheeze in the last 12 months for infants aged 16–24 months old (adjusted OR, 95% CI) Alpha Carotene Quartiles 1. 1.00 2. 0.92 (0.54, 1.55) 3. 1.02 (0.61, 1.72) 4. 1.07 (0.64, 1.80) *p*‐trend 0.70 Beta Carotene Quartiles 1. 1.00 2. 1.14 (0.69, 1.40) 3. 0.85 (0.50, 1.43) 4. 1.06 (0.63, 1.77) *p*‐trend 0.87 Vitamin C Quartiles 1. 1.00 2. 1.03 (0.62, 1.72) 3. 1.05 (0.73, 1.73) 4. 0.98 (0.58–1.66) *p*‐trend 0.97 Vitamin E Quartiles 1. 1.00 2. 0.53 (0.32, 0.88) 3. 0.69 (0.42, 1.13) 4. 0.54 (0.32, 0.90) *p*‐trend 0.04	NOS 7
Nwaru et al. [35] Finland	Cohort	2441 mother–infant pairs	5 years	Asthma	Questionnaire	Vitamin A (alpha and beta carotene) Vitamin C Vitamin E (alpha and gamma tocopherol)	FFQ (8th month of pregnancy)	Asthma–adjusted hazard ratio (95% CI) Vitamin A 1.20 (0.92–1.56) ‐alpha carotene1.18 (0.86–1.62) ‐beta carotene 1.15 (0.84–1.58) Vitamin C 0.92 (0.70–1.22) Vitamin E 1.02 (0.70–1.47) ‐alpha tocopherol 1.02 (0.70–1.47) ‐gamma tocopherol 1.01 (0.76–1.34)	NOS 6
aTurner et al. [36] Scotland	Cohort	1145 pregnant women	5 years	Wheeze & asthma	Questionnaire	Vitamin E (alpha tocopherol)	Plasma levels measured at 1st trimester ultrasound with normal phase HPLC and FFQ (32 weeks gestation)	Quintiles of maternal vitamin E Ever asthma (adjusted OR, 95% CI) 0.84 (0.72, 0.98) *n* = 1018 Doctor‐confirmed asthma (adjusted OR, 95% CI) 0.83 (0.71, 0.97) *n* = 1018 Ever wheeze (adjusted OR, 95% CI) 0.89 (0.78, 1.04) *n* = 1020 Wheeze in last 12 months (adjusted OR, 95% CI) 0.83 (0.71, 0.95) *n* = 1024	NOS 7
West et al. [37] Australia	Cohort	420 pregnant women assessed 300 mother–infant pairs	1 year	Wheeze	Questionnaire	Vitamin A (beta carotene) Vitamin C Vitamin E	FFQ (> 28 weeks gestation)	Wheeze (adjusted OR, 95%CI) Beta‐carotene Quartiles 1: 1.00 2: 0.54 (0.23, 1.22) 3: 1.09 (0.70, 2.88) 4: 0.73 (0.33, 1.61) *p*‐trend = 0.2 Vitamin C Quartiles 1: 1.00 2: 1.11 (0.51, 2.40) 3: 1.33 (0.61, 2.90) 4: 0.40 (0.16, 0.98) *p*‐trend = 0.06 Vitamin E Quartiles 1: 1.002: 0.89 (0.41, 1.93) 3: 0.72 (0.33, 1.41) 4: 0.77 (0.36, 1.66) *p*‐trend = 0.9	NOS 5
Brzozowska et al. [19] Poland	prospective cohort	557 mother‐child pairs	1‐9 years	Wheeze	Health examination and interview	Vitamin A (dietary sources) Vitamin A (from dietary sources and supplementation) Vitamin C (dietary sources) Vitamin C (from dietary sources and supplementation) Vitamin E (dietary sources) Vitamin E (from dietary sources and supplementation)	Maternal and cord blood FFQ meeting EAR vs not meeting EAR during pregnancy	Dietary sources Vitamin A (adjusted OR, 95% CI) Wheeze Age of the child 1 year—0.18 (0.01, 1.04) 2‐ year—0.31 (0.01, 2.53) Vitamin C (adjusted OR, 95% CI) Wheeze Age of the child 1 year—1.11 (0.49, 2.64) 2‐ year ‐ 4.17 (1.48, 13.56) Vitamin E (adjusted OR, 95% CI) Infection Wheeze Age of the child 1 year—0.60 (0.25, 1.55) 2‐ year—0.94 (0.33, 2.87) Dietary sources and supplementation Vitamin A (adjusted OR, 95% CI) Wheeze Age of the child 1 year—0.26 (0.01, 1.47) 2 year—0.31 (0.01, 2.53) Vitamin C (adjusted OR, 95% CI) Wheeze Age of the child 1 year—1.37 (0.61, 3.08) 2 year—2.55 (1.03, 6.63) Vitamin E (adjusted OR, 95% CI) Wheeze Age of the child 1 year—0.60 (0.26, 1.38) 2 year—1.48 (0.55, 4.35)	NOS 7
Litonjua et al. [38] USA	Cohort	1290 mother–infant pairs	2 years	wheeze	Questionnaire	Vitamin A (alpha and beta‐carotene) Vitamin C Vitamin E	FFQ (at approximately 10 and 27 weeks gestation)	Any wheezing in the first 2 years (adjusted odds ratio, 95% CI) Vitamin C quartiles 1: 1.00 2: 0.93 (0.65, 1.35) 3: 0.84 (0.58, 1.22) 4: 0.79 (0.54, 1.15) *p*‐trend = 0.2 Vitamin E quartiles 1: 1.00 2: 0.90 (0.62, 1.31) 3: 0.84 (0.58, 1.22) 4: 0.70 (0.48, 1.03) *p*‐trend = 0.06 α‐Carotene quartiles 1: 1.00 2: 0.87 (0.60, 1.26) 3: 1.00 (0.69, 1.45) 4: 0.92 (0.63, 1.34) *p*‐trend = 0.8 β‐Carotene quartiles 1: 1.00 2: 1.28 (0.88, 1.88) 3: 1.21 (0.82, 1.77) 4: 0.98 (0.66, 1.47) *p*‐trend = 0.9	NOS 6
Hong et al. [20] Republic of Korea	Cohort	983 mother–infant pairs 550 infant outcomes measured	1 year	RTI	Questionnaire	Vitamin A Vitamin C Vitamin E	FFQ (26 weeks gestation)	Risk of respiratory tract infection (adjusted OR, 95% CI) Vitamin A Tertile (T)1: 1.00 T2: 0.88 (0.55, 1.42) T3: 0.69 (0.43, 1.11) Vitamin C T1: 1.00 T2: 0.82 (0.51, 1.32) T3: 0.68 (0.42, 1.09) Vitamin E T1: 1.00 T2: 0.44 (0.27, 0.71) T3: 0.72 (0.44, 1.17)	NOS 7
Hanson et al. [39] USA	Cross‐sectional	180 pregnant women 173 mother–infant pairs	Birth	RDS	Medical Record	Vitamin A (retinol)	Serum retinol by HPLC	Serum retinol category (μmol/L) Newborn with RDS *n* (%) (*p* = 0.005) ≤ 0.7: 1 (4.0%) > 0.7–1.05: 18 (72.0%) > 1.05: 6 (24.0%) Newborn without RDS n (%) ≤ 0.7: 15 (9.9%) > 0.7–1.05: 56 (36.8%) > 1.05: 81 (53.3%)	NOS 8
^a^Tepper et al. [40] USA	RCT	178 mother–child dyads	5 years	Lung function via forced oscillation measurement	Medical assessment	Intervention group: Vitamin C (500 mg/day) Control group: placebo	Plasma ascorbic acid level	Spirometry of placebo versus vitamin C group at 5 years FVC *p*‐value = 0.59008 FEV_1_ *p*‐value = 0.1885	Low RoB
^a^Devereux et al. [6] Scotland	Cohort	1861 pregnant women	5 years;	Ever wheeze & Ever Asthma	Questionnaire	Vitamin E intake and plasma level	p	Ever asthma (adjusted OR, 95% CI) Intake Quintile (Q)1: 1.00 Q2: 0.64 (0.35, 1.16) Q3: 0.60 (0.33, 1.12) Q4: 0.59 (0.32, 1.10) Q5: 0.47 (0.24, 0.92) *p*‐trend = 0.04 Ever wheeze (adjusted OR, 95% CI) Intake Quintile (Q)1: 1.00 Q2: 0.84 (0.52, 1.37) Q3: 0.92 (0.57, 1.50) Q4: 0.50 (0.29, 0.85) Q5: 0.75 (0.44, 1.28) *p*‐trend = 0.07	NOS 7
Gromadzinska et al. [21] Poland	Cohort	252 pregnant women	1 year and 2 years	Wheeze	Questionnaire & paediatrician assessment	Vitamin A (including beta‐carotene), Vitamin E	Maternal plasma levels by high performance liquid chromatography in 1st trimester and at delivery	Wheezing at age 2 years (odds ratio, 95% CI) 1st trimester serum levels of smokers during pregnancy β‐Carotene, µg/mL: 1.56 (0.01–487.3) Vitamin A, µg/mL: 5.18 (0.06–485.0) Vitamin E, µg/mL: 1.11 (0.87–1.41) 1st trimester serum levels of non‐smokers during pregnancy β‐Carotene, µg/mL: 0.02 (0.01–2.18) Vitamin A, µg/mL: 0.47 (0.05–4.19) Vitamin E, µg/mL: 1.00 (0.85–1.16) Serum levels at delivery of smokers during pregnancy β‐Carotene, µg/mL: 16.94 (0.06–465.0) Vitamin A, µg/mL: 1.56 (0.07–36.06) Vitamin E, µg/mL: 1.02 (0.86–1.22) Serum levels at delivery of non‐smokers during pregnancy β‐Carotene, µg/mL: 1.41 (0.07–29.38) Vitamin A, µg/mL: 0.38 (0.05–3.22) Vitamin E, µg/mL: 1.07 (0.96–1.20) Wheezing at age 1 year (odds ratio, 95% CI) 1st trimester serum levels of smokers during pregnancy β‐Carotene, µg/mL: 0.06 (0.01–32.72) Vitamin A, µg/mL: 0.47 (0.01–17.07) Vitamin E, µg/mL: 0.99 (0.77–1.26) 1st trimester serum levels of non‐smokers during pregnancy β‐Carotene, µg/mL: 0.54 (0.01–22.42) Vitamin A, µg/mL: 1.52 (0.17–13.92) Vitamin E, µg/mL: 1.11 (0.94–1.32) Serum levels at delivery of smokers during pregnancy β‐Carotene, µg/mL: 0.20 (0.01–1.50) Vitamin A, µg/mL: 2.68 (0.12–61.09) Vitamin E, µg/mL: 0.91 (0.75–1.09) Serum levels at delivery of non‐smokers during pregnancy β‐Carotene, µg/mL: 0.75 (0.03–20.79) Vitamin A, µg/mL: 0.18 (0.02–1.97) Vitamin E, µg/mL: 1.04 (0.93.1.18)	NOS 8

Abbreviations: CI, confidence interval; FFQ, food frequency questionnaire; HPLC, high‐performance liquid chromatography; NOS, Newcastle‐Ottawa scale; OR, odds ratio; PFT, pulmonary function test; PPROM, preterm prelabour rupture of membranes; QA, quality assessment; RCT, randomised controlled trial; RDS, respiratory distress syndrome; RoB, Risk of Bias; RTI, respiratory tract infection; TPTEF:TE, time to peak tidal expiratory flow as a proportion of total expiratory time.

^a^ These papers report on the same participants, but at different timepoints for the outcome.

#### 4.1.1 Description of Included Studies

In total, six studies were RCTs and 12 were observational studies. Papers were published between 2003 and 2024. Countries included were Poland, Korea, Iran, Tanzania, United Kingdom, United States of America, Canada, Scotland, Denmark, Japan, Finland and Australia.

Vitamin intake was commonly measured by Food Frequency Questionnaire (FFQ) (*n* = 10) and circulating plasma levels (*n* = 8). Papers examined vitamin A (*n* = 11), vitamin C (*n* = 11), vitamin E (*n* = 11) or combined vitamin C and E (*n* = 4). Some studies examined multiple vitamins. Four studies followed up the offspring at birth, one examined the first year of life, two examined the second year of life, one looked at 18 months, four examined 2 years of age and five collected outcome/s at 5 years of age. The mean NOS score for the 12 included cohort studies was 6.75, of which 11 scored ≥ 6 (good or very good quality). All the included RCTs were rated as having low risk of bias (Supporting Information S1).

### 4.2 Maternal Vitamin A and Offspring Respiratory Outcomes

One RCT, nine cohort studies and one cross‐sectional study were identified that assessed maternal vitamin A or vitamin A precursor (beta‐carotene) intake during pregnancy and its association with offspring respiratory outcomes, including wheeze (*n* = 7) [4, 19, 21, 33, 34, 37, 38], asthma (*n* = 1) [35], cough (*n* = 1) [28], RDS (*n* = 1) [39] and RTI (*n* = 1) [20]. Nine of the 10 studies assessed infant outcomes before the age of 2 years, and one assessed asthma at the age of 5 years. Sample sizes ranged from 173 to 44,594 mother–infant pairs.

#### 4.2.1 Wheeze

Six cohort studies assessed infant wheeze within the first 2 years of life as the primary outcome. Miyaki et al. also used a food frequency questionnaire (FFQ), while Gromadzinska et al. and Hanson et al. ascertained maternal vitamin status from serum measurements, while Martindale et al. used both methods [4, 21, 33, 39].

No statistically significant associations between maternal vitamin A status and infant wheeze up to 2 years were found in any study [4, 19, 21, 33, 34, 37, 38]. For example, West et al. analysed 300 Australian mother–infant dyads and found an unadjusted odds ratio for wheeze in the first 12 months of 0.77 (95% CI: 0.38–1.57) for quartile 4 versus quartile 1 of maternal beta‐carotene exposure, derived from an FFQ [37].

#### 4.2.2 Asthma

Two cohort studies were identified that assessed offspring asthma and no significant associations with maternal vitamin A status were found [34, 35]. The study of the Danish National Birth Cohort assessed maternal vitamin A intake with an FFQ and incidence of childhood asthma at 18 months using the International Study of Asthma and Allergies in Childhood (ISAAC) questionnaire [34]_._ No associations were found between total vitamin A intake (diet + supplements) and asthma; however, maternal vitamin A intake from supplements alone was found to have a direct relationship, increasing the odds of asthma by 17% in children whose mothers supplemental intake was in the highest quintile versus the lowest (Q5 vs. Q1, OR: 1.17, 95% CI: 1.05–1.30) [34]. Nwaru et al. also employed FFQs and the ISAAC questionnaires, but did not find any significant associations between maternal vitamin A intake (as a continuous measurement) and asthma at age 4–5 years (unadjusted hazard ratio [HR] 1.22, 95% CI: 0.98–1.53) [35].

#### 4.2.3 Cough

An RCT of vitamin A supplementation (500IUA + 30 mg β‐carotene) for mothers with HIV found a 31% reduced risk (RR: 0.69, 95% CI: 0.49–0.96) of cough with a rapid respiratory rate at 2 years of age versus placebo [28].

#### 4.2.4 RDS

The study from Hanson et al. measured serum retinol in mothers at the time of birth and showed no significant association between maternal retinol category (deficient, insufficient, adequate) and infant RDS after adjusting for gestational age and smoking [39].

#### 4.2.5 RTI

One study examined the risk of RTI across three tertiles of maternal vitamin A intake, determined by FFQ at 26 weeks of gestation. While there was a reduced risk of RTI with increasing levels of vitamin intake, this did not reach statistical significance [20]. Due to the dissimilarity of many factors including infant age and differences in exposure and outcomes measured, meta‐analysis could not be performed for maternal vitamin A.

### 4.3 Maternal Vitamin C and Offspring Respiratory Outcomes

Eight cohort studies [4, 19, 20, 29, 33, 35, 37, 38] and two RCTs [15, 31, 32, 40] assessed the association between maternal vitamin C intake in pregnancy and offspring respiratory health, with sample sizes ranging from 178 to 2441. Eight studies reported on wheeze [4, 15, 19, 29, 31, 33, 37, 38], two studies reported on asthma [29, 35], three on lung function [15, 31, 40] and one study reported on RTI [20].

#### 4.3.1 Wheeze

Seven cohort studies assessed wheeze up to 2 years of age [4, 19, 20, 29, 33, 35, 37, 38], with different studies having slightly different age ranges (e.g., 13–24 months, 0–12 months). Nwaru et al. reported asthma at 5 years [35] and Brzozowska et al. reported asthma up to 9 years of age [19, 35]. Studies divided maternal vitamin C intake into tertiles, quartiles or quintiles. When odds ratios for wheeze were adjusted for confounders, two studies found an increased odds of wheeze at approximately 2 years with increasing vitamin C intake [19, 33], while four studies found no association between wheeze and maternal vitamin C intake (Table 1) [4, 29, 37, 38]. McEvoy et al. found a reduced odds of wheeze at 12 months of age in the intervention arm across several sites; however, only when supplementation commenced ≤ 18 weeks of gestation [15]. A 2023 follow‐up paper from the 2020 RCT from McEvoy et al. reported that children of pregnant smokers who were randomised to the vitamin C arm during pregnancy had a significant reduction in wheeze at the age of 5 years (aOR: 0.41, 95% CI: 0.23–0.74) [40].

#### 4.3.2 Meta‐Analysis

**Figure 2:**
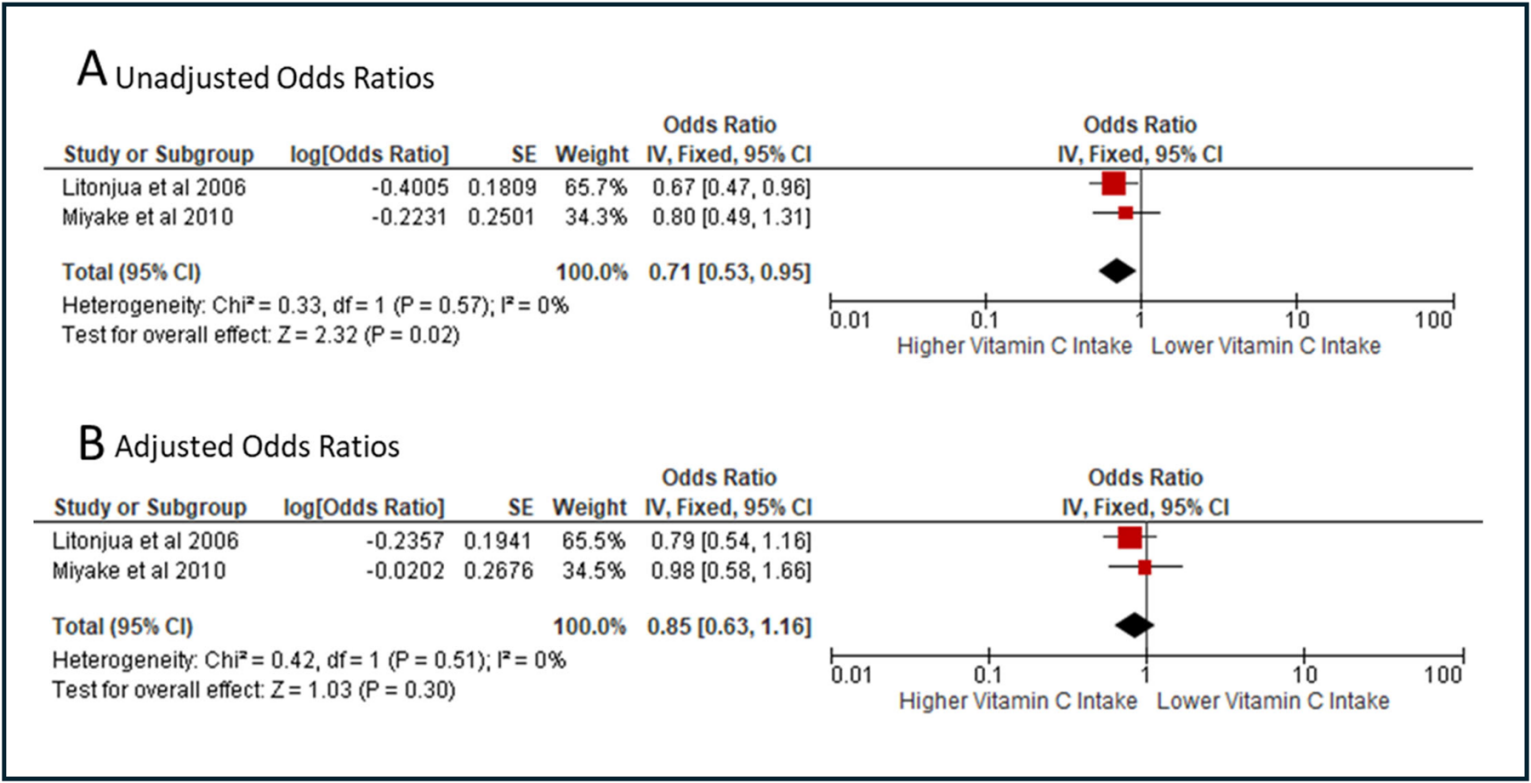
Meta‐analysis of studies examining the relationship between maternal vitamin C intake during pregnancy and wheeze at 2 years of age. Panel A shows unadjusted results, panel B shows adjusted results. Adjusted Odds Ratio—Litonjua et al. was adjusted for birth weight, neonate sex, maternal age, maternal pre‐pregnancy body mass index, breastfeeding duration, the number of children < 12 years old in the home, postnatal passive smoke exposure, family income and maternal and paternal asthma. Miyaki et al. was adjusted for maternal age, gestation at baseline, residential municipality at baseline, family income, maternal and paternal education, maternal and paternal history of asthma, atopic eczema and allergic rhinitis, changes in maternal diet in the previous 1 month, season when data at baseline were collected, maternal smoking during pregnancy, baby's older siblings, baby's sex, baby's birth weight, household smoking in the same room as the infant, breastfeeding duration and age of infant.

We performed a meta‐analysis combining the unadjusted odds ratios from Litonjua et al. and Miyake et al., finding a 29% decreased odds (OR: 0.71, 95% CI: 0.53‐0.95, *I* ^2^ = 0%) for wheeze at 2 years of age among children whose mothers had a higher vitamin C intake during pregnancy (highest quartile vs. the lowest quartile) (Figure 2A). However, when using the adjusted ORs from these studies, the result was no longer significant (OR: 0.85, 95% CI: 0.63–1.16, *I* ^2^ = 0%, Figure 2B) [4, 38]. The certainty of evidence of this meta‐analysis was rated as very low using GRADE, due to the included studies being observational, and imprecision, with data coming from only two studies.

#### 4.3.3 Asthma

Maternal total and dietary vitamin C intake during pregnancy was not associated with asthma in one study (adjusted hazard ratio [HR] 0.92, 95% CI: 0.70–1.22) [35], while an RCT showed no influence of maternal vitamin C and E supplementation on asthma symptoms in the first 12 months of life compared with placebo (OR: 0.94, 99% CI: 0.42–2.11) [39].

#### 4.3.4 Lung Function

Two RCTs by McEvoy et al. randomised pregnant women (enroled at 13–23 weeks gestation) who were current smokers to receive supplementation with vitamin C (vs. placebo) until birth. The 2014 study found that the newborns of women randomised to vitamin C (560 mg/day, *n* = 76) had improved pulmonary function at 72 h of age, compared with those randomised to placebo (60 mg/day antenatal supplement, *n* = 83). Newborn lung function was assessed during quiet sleep, with measures collected during tidal breathing. Where a minimum of 50 tidal breathing flow‐volume loops were available with inspiratory and expiratory volumes within 15%, the authors calculated tidal volumes and the time to peak tidal expiratory flow to expiratory time (TPTEF:TE ratio). The single‐breath occlusion technique with a minimum of 10 breaths was used to assess passive respiratory system compliance (Crs), with Crs normalised for body weight (Crs/kg). For a sub‐group of infants, nitrogen washout was used to assess functional residual capacity (FRC). The measurement of TPTEF:TE (*p* = 0.006) and passive respiratory system compliance/kg (Crs/kg, *p* = 0.0012) was higher in the intervention versus placebo group, respectively [31]. In the 2020 trial, infants of smoking mothers allocated to vitamin C supplementation (500 mg/day) versus placebo (no supplementation) during pregnancy had significantly increased forced expiratory flows (FEFs) for FEF_75_ (75% of forced vital capacity) at 12 months of age compared with those allocated to placebo (*p* = 0.025) [15]. Measurements were made in infants under chloral hydrate sedation, using the raised volume rapid thoracic compression technique, with three technically acceptable curves with FEF25‐75 and FVC within 10% required [15]. Tepper et al., who followed up the same cohort at 5 years of age, found no difference between lung function via forced oscillation technique (FVC *p*‐value = 0.59008 and FEV_1_ *p*‐value = 0.1885) based on placebo versus intervention group [40].

#### 4.3.5 RTI

Similar to their findings for vitamin A intake at 26 weeks of gestation, Hong et al. found no statistically significant influence of vitamin C intake on RTI in offspring at 1 year of age [20].

### 4.4 Maternal Vitamin E and Offspring Respiratory Outcomes

Ten cohort studies measured maternal vitamin E intake and the respiratory health of the offspring. All were cohort studies with sample sizes ranging from 420 to 44,594. Nine studies reported on wheeze [4, 6, 19, 21, 33, 34, 36, 37, 38], three reported asthma [6, 35, 36], one study measured lung function by spirometry [6] and one study measured RTI [20].

#### 4.4.1 Wheeze

Nine cohort studies were identified that assessed wheeze outcomes in offspring [4, 6, 19, 21, 33, 34, 36, 37, 38]. Martindale et al. recorded 'wheeze ever' in the second year of life and found reduced odds for wheeze in the absence of a cold for the highest vitamin E quintile versus the lowest (aOR: 0.47, 95% CI: 0.25–0.86). This effect remained when adjusting for vitamin C intake and known confounders. However, when examining more broadly ‘wheeze ever’ in the first 2 years of life, there was no association across the quintiles [33]. Devereux et al. followed up the same cohort at age 5 years and found significant negative associations between maternal vitamin E and wheeze in the previous year (OR: 0.79, 95% CI: 0.65–0.95), wheeze in the absence of a cold (OR: 0.82, 95% CI: 0.71‐0.95), and persistent wheezing (OR: 0.77, 95% CI: 0.63–0.93) [6]. Gromadzinska et al. did not find any significant association between the mean levels of vitamin E in plasma (first trimester or at delivery) and wheeze when comparing 'healthy children' to those who wheezed in either their first or second year of life [21]. West et al. followed infants to 12 months of age and found no significant association between the quartiles of maternal vitamin E intake and wheeze, whether unadjusted or adjusted for confounders [37]. Nwaru et al. conducted a follow‐up at age 5 and found no significant associations between maternal vitamin E intake and wheeze in the previous 12 months [35].

#### 4.4.2 Meta‐Analysis

**Figure 3:**
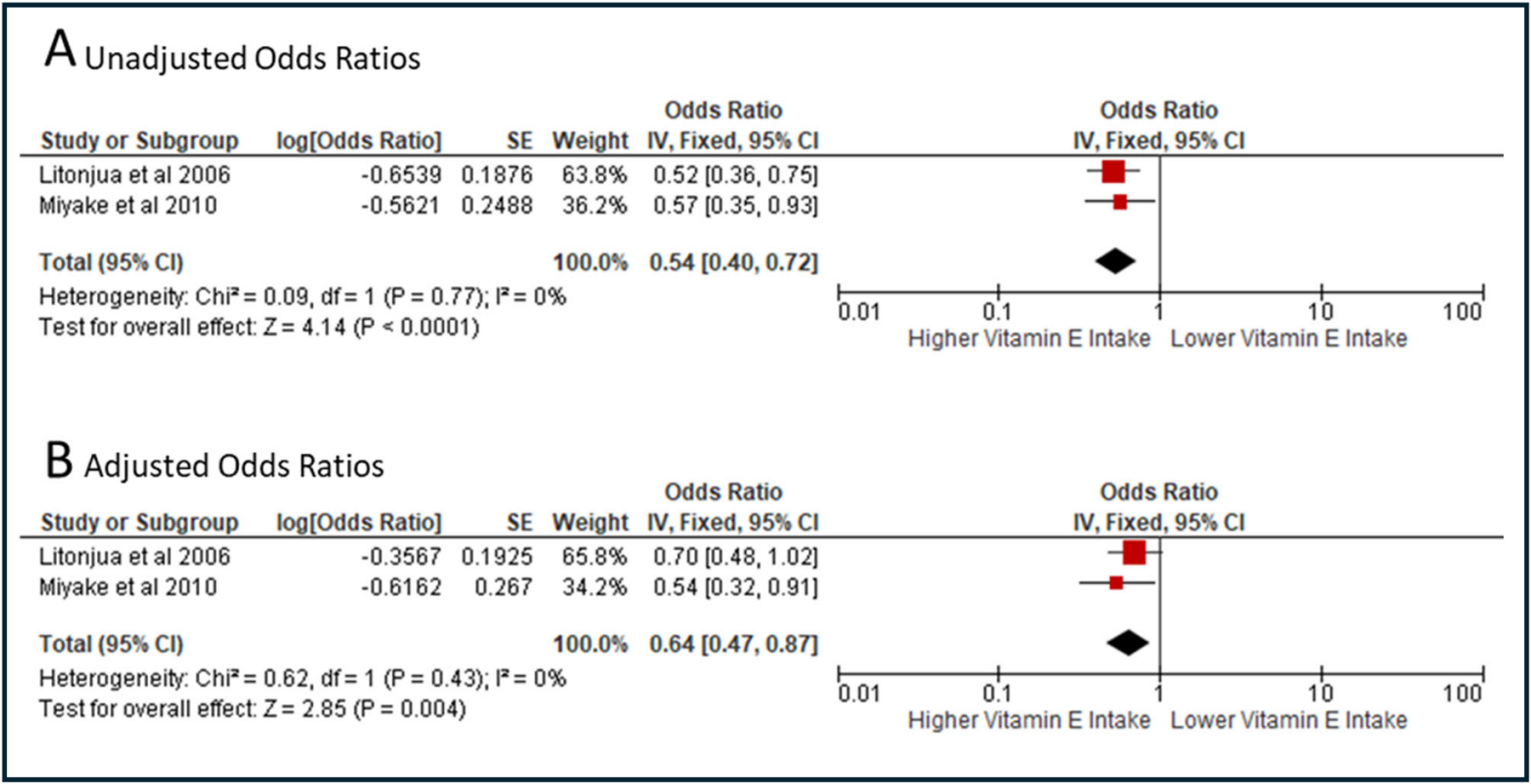
Meta‐analysis of studies examining the relationship between maternal vitamin E Intake during pregnancy and wheeze at 2 years of age. Panel A shows unadjusted results, panel B shows adjusted results. Adjusted Odds Ratio—Litonjua et al. was adjusted for birth weight, neonate sex, maternal age, maternal pre‐pregnancy body mass index, breastfeeding duration, the number of children < 12 years old in the home, postnatal passive smoke exposure, family income and maternal and paternal asthma. Miyaki et al. were adjusted for maternal age, gestation at baseline, residential municipality at baseline, family income, maternal and paternal education, maternal and paternal history of asthma, atopic eczema and allergic rhinitis, changes in maternal diet in the previous 1 month, season when data at baseline were collected, maternal smoking during pregnancy, baby's older siblings, baby's sex, baby's birth weight, household smoking in the same room as the infant, breastfeeding duration and age of infant.

Meta‐analysis of two cohort studies (Litonjua et al. and Miyake et al.) which recorded wheeze in offspring up to 2 years of age showed that maternal vitamin E intake in the highest quartile versus the lowest was associated with significantly reduced odds of wheeze in the offspring (OR: 0.54, 95% CI: 0.40‐0.72, *I* ^2^ = 0% Figure 3A) [4, 38]. This association remained significant when adjusted data were used (aOR: 0.64, 95% CI: 0.47–0.87, *I* ^2^ = 0% Figure 3B), with no heterogeneity between studies. The certainty of evidence of this meta‐analysis was rated as very low using GRADE, due to the included studies being observational, and imprecision, with data coming from only two studies.

#### 4.4.3 Asthma

Three studies were identified that assessed asthma as the outcome [6, 35, 36]. Devereux et al. assessed quintiles of maternal vitamin E intake and ever‐asthma, doctor‐confirmed asthma and asthma and wheeze in the last 12 months at age 5 years. Results indicated higher quintiles of vitamin E (3rd, 4th and 5th) relative to the lowest quintile intake during pregnancy were associated with a lower odds of wheeze, doctor‐diagnosed asthma and asthma ever, after adjustment for confounders [6]. Turner et al. also found higher maternal vitamin E quintiles (3rd, 4th, 5th) were associated with lower odds of asthma ever (OR: 0.84, 95% CI: 0.72–0.98) and doctor diagnosed asthma (OR: 0.83, 95% CI: 0.71–0.97), however, when the model was adjusted for crown‐rump length, these all became nonsignificant [36]. Nwaru et al. did not find any significant association between maternal vitamin E intake and the risk of asthma in the offspring at 5 years [35].

#### 4.4.4 Lung Function

One study measured lung function using spirometry in offspring at age 5 years [6]. Maternal vitamin E (α‐tocopherol) concentration in plasma at 12 weeks gestation (but not maternal vitamin E Intake measured by FFQ at 32 weeks) was positively associated with both pre and post‐bronchodilator forced expiratory volume at 0.5 s (FEV_0.5_), pre‐ and post‐bronchodilator FEV_0.75_ and pre‐ and post‐bronchodilator forced vital capacity (FVC), as well as post‐bronchodilator FEV_1_ [6]. In multivariate linear regression, including potential confounders such as height, weight and sex, children whose mothers were in the highest versus the lowest tertile of plasma alpha‐tocopherol had an increase in post‐bronchodilator FEV_1_ of 77 mL [6].

#### 4.4.5 RTI

Offspring RTI risk was significantly reduced in mothers with vitamin E intake in the second tertile compared with the first tertile; however, there was no difference in those with the highest vitamin E intake (third tertile) compared with the lowest intake [20].

### 4.5 Maternal Vitamin C and E Supplementation and Offspring Respiratory Outcomes

#### 4.5.1 Wheeze

The trial from Greenough et al. did not show any association between vitamin C and E supplementation and wheeze in the first 2 years of life [29].

#### 4.5.2 Respiratory Distress Syndrome (RDS)

Two RCTs assessed a combination of vitamin C and E supplementation versus placebo and infant RDS as an outcome [27, 29, 30]. In both RCTs, the population was infants who were born pre‐term; in one study, women also had premature rupture of membranes [27] and in a second study, mothers were also at risk for pre‐eclampsia [29]. Borna et al., who used a vitamin C dose of 500 mg/day and vitamin E 400IU/day versus placebo, found that the incidence of infant RDS was not significantly different between the two groups (*p* = 0.27) [27].

#### 4.5.3 Meta‐Analysis

**Figure 4:**
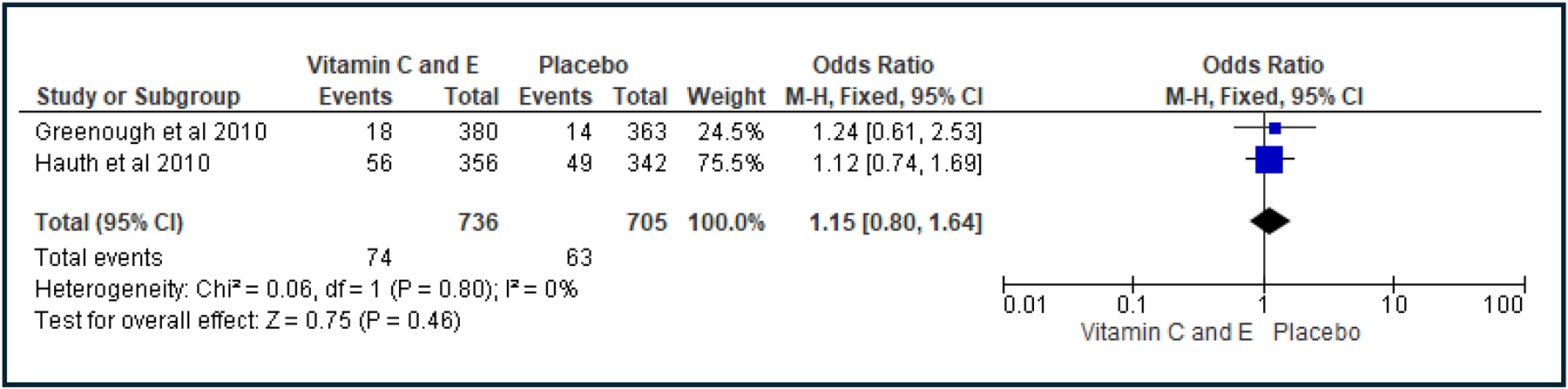
Unadjusted meta‐analysis of studies examining the relationship between Vitamin E and C supplementation versus placebo on infant respiratory distress syndrome (RDS).

Two trials with the same supplement dose (vitamin C 1000 mg/day and vitamin E 400 IU/day) from 9 to 16 weeks of gestation were combined in a meta‐analysis to examine the odds of infant RDS [29, 30]. Vitamin supplementation was not associated with infant RDS (OR: 1.15, 95% CI: 0.80–1.64, *I* ^2^ = 0%) relative to placebo (Figure 4). The certainty of evidence of this meta‐analysis was rated as moderate using GRADE, due to the included studies being RCTs with low risk of bias, but with data coming from only two studies.

## 5 Discussion

This is the first systematic review and meta‐analysis to examine the associations between maternal biomarkers or dietary or supplemental intake of vitamins A, C and/or E during pregnancy and offspring respiratory outcomes up to the age of 5 years. There was no evidence to suggest maternal vitamin A intake improves early life respiratory outcomes in offspring. Vitamin C intake had mixed evidence with some studies showing some benefit for infant respiratory health in the context of maternal smoking when supplementation commences early in gestation, and others suggesting no benefit. Higher intake of vitamin E during pregnancy was shown to be associated with a lower odds of adverse respiratory health outcomes (wheeze and asthma ever) in offspring at ages 2 and 5 years. A meta‐analysis with a moderate certainty demonstrated no benefit of vitamin C and vitamin E supplementation together during pregnancy for infant respiratory distress syndrome.

There was no evidence of an association between maternal vitamin A and offspring wheeze or asthma across eight cohort studies. Only Fawzi et al.'s randomised, double‐blinded, placebo‐controlled trial for HIV‐1 positive women had a positive finding, showing a 31% risk reduction for developing a cough with rapid respiratory rate during their first 2 years of life [28]. Children born to HIV‐positive mothers are susceptible to micronutrient deficiencies, and consequently, they receive 6‐monthly vitamin A doses as part of the standard of care [41]. The results of this study suggest that where there is a deficiency, vitamin A supplementation may be associated with improved cough in children [28]. These findings are particularly pertinent to low and middle‐income countries where vitamin A deficiency remains a significant health problem [9].

Our review included three trials demonstrating that vitamin C supplementation to pregnant women who are unable to quit smoking improves the lung function of their infants [15, 31, 32]. As vitamin C supplementation is generally well tolerated, cost‐effective and safe, this could prove to be an effective intervention. Studies have shown that babies of mothers who smoked during pregnancy are far more likely to have adverse health outcomes throughout their lives [15, 42, 43], with respiratory function also impaired, and decreased airway function already evident from birth, when compared with babies of nonsmoking mothers [15]. Furthermore, there is an increased risk that these infants will develop wheeze and asthma later in life [43]. Despite these outcomes, more than half of the women who smoke will continue to do so throughout their pregnancy [15, 43]. As many smoking cessation trials have been ineffective, vitamin C supplementation has been proposed as a way to improve health outcomes for these children. Further studies are required to explore if vitamin C may also convey protection beyond maternal smoking in other scenarios that induce oxidative stress in pregnancy, such as high air pollution exposure.

The majority of studies assessing vitamin C were cohorts and cross‐sectional studies, and all used FFQs to estimate maternal intake. We performed a meta‐analysis combining the unadjusted odds ratios from Litonjua et al. and Miyake et al., finding a 29% decreased odds for wheeze at 2 years of age among children whose mothers had a higher vitamin C intake during pregnancy (highest quartile vs. the lowest quartile), however, using the adjusted ORs from these studies the result was no longer significant [4, 38]. Most studies reported either a slight or no significant benefit with higher vitamin C intake, with two studies reporting increased odds of wheeze in the second year of life with higher vitamin C intake [19, 33]. This may be a surprising result as previous studies in adults have found mostly beneficial associations between vitamin C, fruit intake, asthma and respiratory symptoms [14, 44]. Although fruit intake is generally associated with better rather than worse self‐reported health in adults, it is possibly the result of reverse causation, with mothers that have respiratory symptoms themselves consuming higher amounts of supplements and vitamins, including vitamin C, for preventative measures, leading to a spurious positive association between vitamin C intake and wheeze. This result was seen in the US Nurses' health study, with reverse causation suggested [45]. In addition, the positive association between maternal vitamin C intake and wheeze was not seen with wheeze in the absence of a cold, which may be a better indicator of wheeze with an asthmatic aetiology as opposed to virus‐associated wheeze [33]. Further evidence that the observed association between vitamin C and wheeze may not be a true biological effect is provided by the finding that in a subsample of 223 of these children, cord blood responses to in vitro stimulation with allergens were inversely associated with maternal vitamin E intake but showed no association with vitamin C intake [46]. Our meta‐analysis showed a reduced odds of wheeze when unadjusted data were used; however, after adjustment, the results were no longer significant. Common factors that were adjusted for included maternal age, maternal and paternal asthma, family income, infant sex, passive smoke exposure and family income [4, 38]. Overall, the current data suggest there is unlikely to be any benefit of maternal vitamin C intake during pregnancy for offspring wheeze, and that any effects of this single nutrient may not be as great as the combined effects of identified confounders. However, meta‐analyses were only possible with two studies that reported the same outcomes in the same age range, and we were unable to pool any studies that examined circulating blood levels; therefore, further studies are required to examine the effect of differing dose levels and optimal dietary intake. We were also unable to pool any studies that examined outcomes in children older than 2 years.

Vitamin E displayed the strongest evidence for a positive effect of maternal antioxidant vitamin intake on offspring respiratory outcomes up to 5 years of age. Vitamin E outcomes were reported on most often out of the antioxidants analysed in this review, with data from three RCTs and 10 cohort studies included. Meta‐analysis of two of the cohort studies showed a significant 36% reduction in the odds of wheeze in offspring up to 2 years of age for mothers with the highest quartile of maternal vitamin E intake versus the lowest quartile, as determined by FFQ [4, 38]. Overall, eight out of the 10 studies found that increased maternal vitamin E intake during pregnancy was associated with an improvement in child respiratory outcomes such as wheezing in the previous year, wheezing in the absence of a cold, consulting a doctor for wheeze, asthma, RTI and increased lung function [6]. These findings strongly suggest that maternal vitamin E intake may be protective against developing wheeze and/or asthma during early life, with the quality of most studies being NOS score ≥ 6 indicating good quality evidence.

Current dietary recommendations for vitamin E intake in pregnancy are the same as women who are not pregnant of the same age range (7–8 mg/day α‐tocopherol equivalents/day with an upper limit of 300 mg/day); however, the recommendation during lactation is higher than this [47]. Although supplementation of vitamin E may lack any benefit in regard to perinatal outcomes assessed in a 2015 Cochrane Review [48], the evidence of a protective effect for offspring respiratory health with higher quartiles may warrant further investigation into the benefits of maternal intake of vitamin E‐rich whole foods during pregnancy and exploration of the mechanisms involved.

Quality assessment for the observational studies in this review suggested a medium quality of evidence, with the majority of included studies of good quality (scoring at least 6 by NOS). The cohort and cross‐sectional studies most often used FFQs for gathering exposure data, which was a limitation. Although FFQs are cost‐effective and convenient, they may underrepresent supplement intake as well as introduce recall bias and have been shown to be inferior to other methods such as quantitative 7‐day diaries and 4‐day food records [49, 50, 51]. All included RCTs in this review were of low risk of bias. The use of placebo control groups in most studies, systematic interventions and participant blinding gave these studies increased reliability. Using GRADE, the certainty of the meta‐analyses was rated as very low for the effect of maternal vitamin C or maternal vitamin E on infant wheeze, and moderate for the lack of effect of maternal vitamin C and E supplementation on infant RDS.

Our review had several limitations, including only including studies published in English, and the search being conducted only in four databases. In addition, we were unable to perform large meta‐analyses due to the variability of both exposure metrics and outcomes between studies. In addition, the use of parent report in some studies may impact on the validity of the outcomes in regard to the subjective nature of the data collection and recall bias. In some studies, data collection also occurred retrospectively, which also may increase the chances of recall bias.

## 6 Conclusion

In conclusion, there was no evidence of any association between maternal dietary intake of vitamins A and C with respiratory outcomes in early life. However, vitamin C supplementation during pregnancy in the context of maternal smoking reduced the risk of wheeze and asthma and improved lung function in early life. This systematic review suggests that there may be some benefit to higher maternal vitamin E intake during pregnancy in reducing infant wheeze.

Future research priorities should focus on whether high‐dose maternal supplementation of vitamins A, C and E in the presence of deficiency improves childhood respiratory outcomes.

## Author Contributions

**Vanessa E. Murphy, Adam Collison:** creation of study and developed the study methodology. **Vanessa E. Murphy, Adam Collison, Megan E. Jensen, Vanessa E. Murphy:** acquired the study resources. **Vanessa E. Murphy, Adam Collison:** supervised the research. **Jake Gregson, Farihah Islam, William Huang, Katie Aistrope, Soriah Harvey, Tesfalidet Beyene:** Data collection and analysis. **Jake Gregson, Farihah Islam, William Huang, Katie Aistrope, Soriah Harvey, Tesfalidet Beyene:** formally reviewed and analysed the data. **Vanessa E. Murphy, Soriah Harvey, Tesfalidet Beyene, Jake Gregson, Farihah Islam, William Huang, Katie Aistrope:** wrote the manuscript draft. **Soriah Harvey, Tesfalidet Beyene, Megan E. Jensen, Adam Collison:** reviewed and edited the manuscript; and all authors: read and approved the final manuscript.

## Conflicts of Interest

The authors declare no conflicts of interest.

## 1 Peer Review

The peer review history for this article is available at https://www.webofscience.com/api/gateway/wos/peer-review/10.1111/jhn.70086.

## Supporting information

ROB2 IRPG beta v9 ROB Supplement material.

## Data Availability

The data that support the findings of this study are available from the corresponding author upon reasonable request.
