# Supplementary material for: Vitamin A, C and/or E Intake During Pregnancy and Offspring Respiratory Health: A Systematic Review and Meta‐Analysis
Source: J Hum Nutr Diet. 2025 Jul 2;38(4):e70086. doi: 10.1111/jhn.70086 (PMC12222920; doi:10.1111/jhn.70086)
Supplement: Supplementary file 1 — ROB2 IRPG beta v9 ROB Supplement material. [file JHN-38-0-s001.docx]

Supplementary Material- S1. RoB Cohrane Tool
